# Topical vascular organoid therapy promotes microvascular regeneration and functional recovery in porcine ischemic cardiomyopathy

**DOI:** 10.1016/j.stemcr.2026.102999

**Published:** 2026-07-09

**Authors:** John A. Farag, Shin Yajima, Yujiro Kawai, Koji Kawago, Eric Pfrender, Umayr Syed, Jennifer K. Lyons, Tsuyoshi Ueyama, Hiroyuki Takashima, Yuka Matsuura, Gentaro Ikeda, Yu Liu, Yuanjia Zhu, Stefan Elde, Phillip C. Yang, Y. Joseph Woo, Yasuhiro Shudo

**Affiliations:** 1 Department of Cardiothoracic Surgery, Stanford University School of Medicine, Stanford, CA, USA; 2 Stanford Cardiovascular Institute, Stanford University School of Medicine, Stanford, CA, USA; 3 Department of Surgery, Stanford University School of Medicine, Stanford, CA, USA; 4 Department of Medicine, Division of Cardiovascular Medicine, Stanford University School of Medicine, Stanford, CA, USA; 5 Department of Bioengineering, Stanford University School of Medicine, Stanford, CA, USA

**Keywords:** ischemic heart disease, angiogenesis, smooth muscle cell, reperfusion injury, vascular organoid

## Abstract

Ischemic heart disease (IHD) often progresses to heart failure due to persistent microvascular dysfunction not corrected by conventional epicardial revascularization. We developed a scaffold-free vascular organoid composed of human endothelial progenitor cells (EPCs) and mesenchymal stem cell-derived smooth muscle cells (SMCs) and evaluated its therapeutic potential in a porcine IHD model. Fourteen days after ischemia induction, bilayer EPC-SMC organoids were transplanted onto the left ventricular surface, and animals were followed for four weeks. Cardiac MRI demonstrated the preservation of left ventricular ejection fraction and modest improvement in regional function compared with controls. Histological and immunohistochemical analyses revealed the migration of transplanted cells into host myocardium, increased vascular density, and enhanced vessel maturation, while gene expression profiling showed the upregulation of angiogenesis-related genes. These findings demonstrate that scaffold-free EPC-SMC vascular organoids can engraft, promote microvascular remodeling, and preserve cardiac function after ischemic injury.

## Highlights

• Novel scaffold-free EPC/SMC vascular organoids were generated
• Improved LV function in a porcine ischemia-reperfusion model
• Enhanced engraftment, vascular density, and vessel maturation
• Upregulated angiogenesis-related genes after transplantation

## Graphical abstract

**Figure:**
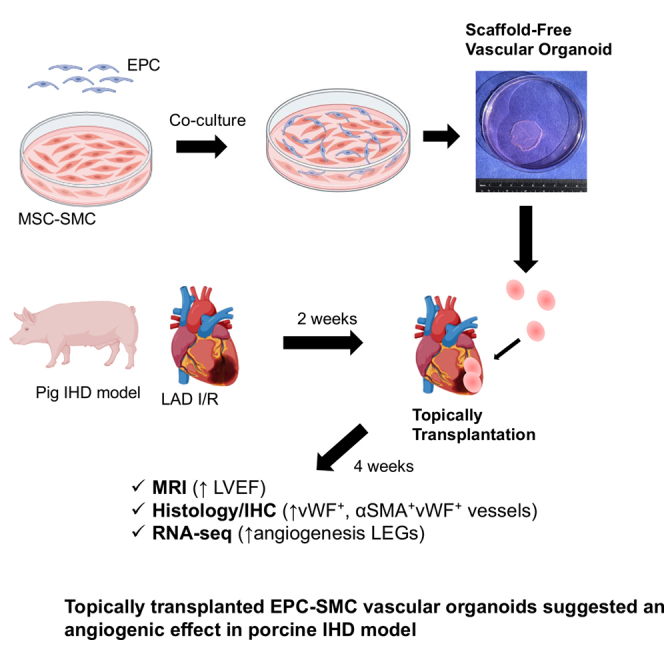

## Teaser

In this study, Yasuhiro Shudo and colleagues demonstrate that scaffold-free EPC/SMC vascular organoid transplantation improves left ventricular function in a porcine ischemia-reperfusion model. This approach enhances engraftment, increases vascular density, and promotes vessel maturation, accompanied by the upregulation of angiogenesis-related genes, highlighting its therapeutic potential for ischemic heart disease.

## Introduction

Coronary artery disease (CAD), which affects approximately twenty million Americans, is the leading cause of death and morbidity in the USA, with an increasing incidence rate (Benjamin et al., 2019). The most severe form of CAD, ischemic heart failure (IHF), affects seven million Americans, with increasing prevalence. With over 400,000 deaths per year attributed to IHF, the five-year mortality rate is 46%. CAD can manifest as an acute myocardial infarction (MI), which can be treated with procedures, such as percutaneous coronary intervention (PCI) and coronary artery bypass grafting (CABG) (Liga et al., 2023). Even with macro-revascularization after MI, nearly 40% of patients progress to IHF (Weir et al., 2006) due to continued deterioration secondary to untreated microvascular injury (Bolognese et al., 2004). There is currently no therapy for microvascular malperfusion, highlighting a critical gap in clinical care and creating an opportunity for innovative therapies to improve heart failure management.

Microvascular malperfusion, which leads to cardiomyocyte dysfunction and injury, ventricular remodeling, and progressive functional deterioration (Robich et al., 2011; Leviner et al., 2018), is an active area of heart failure research. As part of efforts to identify an effective therapy, exosome- (Saha et al., 2019), cytokine- (MacArthur et al., 2013), stem cell- (Hong et al., 2013), and tissue engineering- (Cohen et al., 2014) based micro-revascularization and myocardial repair have been at the forefront of investigation. Cellular therapy is of particular interest because several cell lines have been implicated in angiogenesis and myocardial repair (Saha et al., 2019; Gallet et al., 2015; Suzuki et al., 2021; Atluri et al., 2010; Otto et al., 2012). Many of these therapies have shown initial experimental promise but face limitations due to suboptimal delivery platforms. For example, systemic and transcoronary injection fails to achieve cell localization (Assmus et al., 2006; Schächinger et al., 2006), direct injection is subject to venous washout and introduces a risk of arrhythmogenicity and neoplasm (Dohmann et al., 2005; Van Den Akker et al., 2017), and scaffolded topically transplanted patches carry a risk of constrictive physiology, toxicity, and immune rejection (Cohen et al., 2014; Mei and Cheng, 2020; Guruswamy Damodaran and Vermette, 2018). Additionally, cell survival when integrated into a scaffold is not guaranteed, necessitating an innovative strategy to deliver cells in an organized manner (Yu et al., 2017).

We opted to deliver primary effectors of synergistic microrevascularization (Atluri et al., 2010; Assmus et al., 2006; Schächinger et al., 2006; Hiesinger et al., 2011; Frederick et al., 2010) via a scaffold-free angiogenic vascular organoid. Of particular interest for microangiogenesis, endothelial progenitor cells (EPCs) have high proliferative capacity and the ability to form vascular networks *in vivo* (Kawamoto et al., 2002); however, they do not reach full angiogenic potential due to inadequate support. Mesenchymal stem cells (MSCs), which can differentiate into multiple lineages, have been used for myocardial repair via paracrine signaling and are currently under clinical trials for treating ischemic heart disease (IHD) by exploiting their homing abilities (Perrotta et al., 2020; Squillaro et al., 2016). However, rather than relying on a single agent, our group investigated the synergistic application of EPCs and smooth muscle cells (SMCs) to maximize angiogenic potency (Shudo et al., 2013, 2017). Leveraging the plasticity of MSCs, we developed a methodology to modulate their phenotype into that of SMC-like cells (Suzuki et al., 2010; Shudo et al., 2016), which enhanced their paracrine potency and created an endothelial-mural cell relationship necessary for mature blood vessel formation (Shin et al., 2022). By engineering a scaffold-free vascular organoid comprising EPCs and SMCs, we recreated the native architecture of the mature vasculature by spatially orienting cells in a bilayer, thereby promoting the temporally appropriate distribution of cells into tissue (Shudo et al., 2013, 2017; Shin et al., 2022; Kawamura et al., 2017).

To ensure the translatability of this vascular organoid, we utilized phenotypically modulated MSCs (Shudo et al., 2016), which are readily available from donor bone marrow rather than human SMCs, which must be procured directly from aortic tissue (Shudo et al., 2013). EPCs are purified from donor blood (Masouleh et al., 2010), which allows the banking of cell lines until transplantation.

Having successfully completed xenogenic transplantation of the human-derived vascular organoids into rodents with excellent results (Shin et al., 2022), we aimed to translate our work to an ischemia-reperfusion injury model rather than an acute MI model, as porcine models simulate near-human myocardial thickness and also allow for a more accurate recreation of a post-coronary artery revascularization chronic IHF model. We, therefore, hypothesized that the topical transplantation of SMC-EPC-based vascular organoids into the ischemic porcine heart would limit the functional and geometric deterioration observed in IHF via neoangiogenesis by human cell engraftment, enhance autoangiogenesis by bolstering paracrine signaling, and improve cardiomyocyte survival by enhancing oxygenation.

By successfully proving this hypothesis, we conclude that angiogenic vascular organoid transplantation can serve as an adjunct to existing therapies to decrease IHF morbidity after coronary artery revascularization. Additionally, this therapy may prove to be the first interventional treatment for patients with IHD who are not eligible for PCI or CABG.

## Results

### Creation of SMC-EPC-based vascular organoid

**Figure 1:**
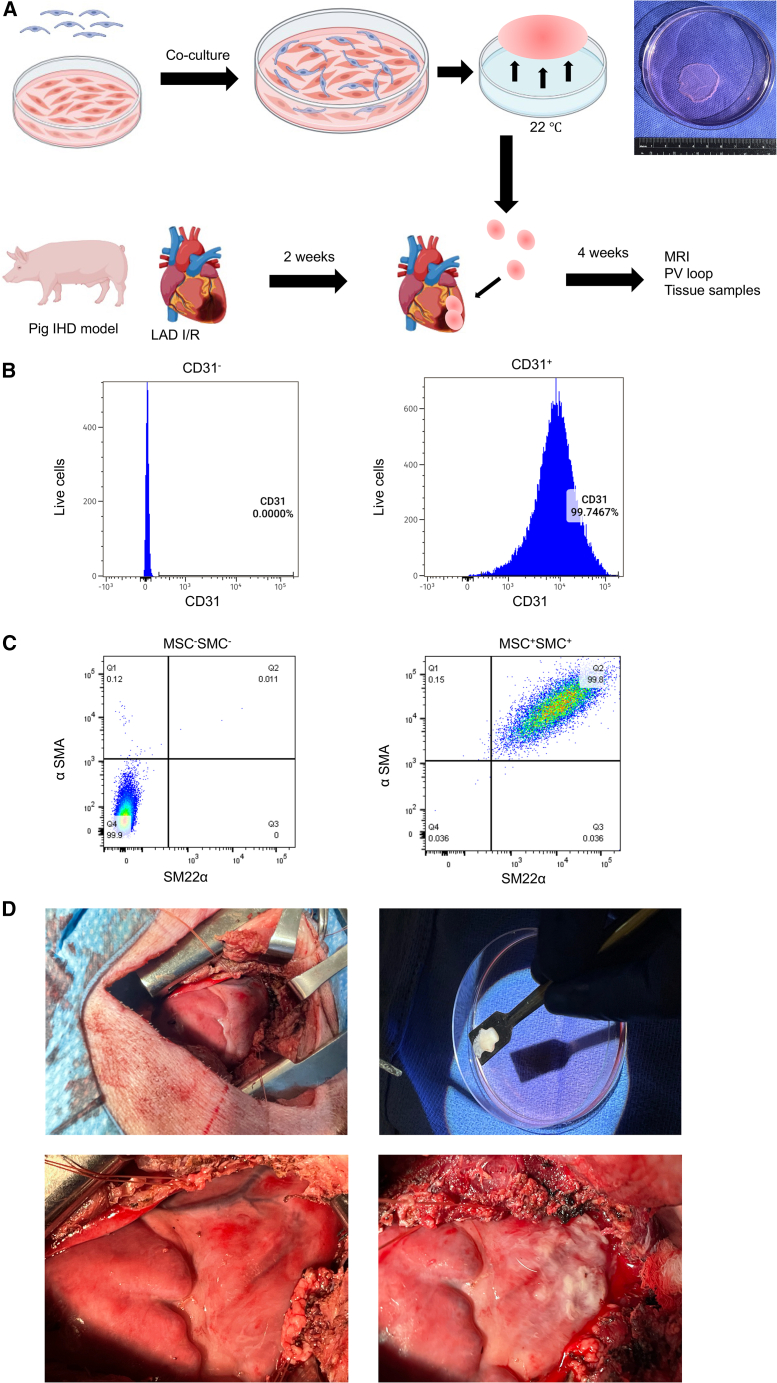
Generation and delivery of SMC-EPC-based vascular organoids (A) Graphical summary of the study. Vascular organoids generated using EPCs and SMCs were transplanted into the ischemic heart disease model pig. (B) Flow cytometry for CD31 on cultured EPCs. (C) Flow cytometry for αSMA and SM22α on cultured SMCs. (D) Transplantation of EPC-SMC-based vascular organoid into the pig via left thoracotomy.

The schema of the study design and creation of the SMC-EPC-based vascular organoid is illustrated in Figure 1A. Vascular organoids were created in an enzyme-free temperature-responsive culture dish. Upon decreasing the temperature from 37°C to 20–22°C, the vascular organoids spontaneously detached from the culture dish (Figure 1A). The engineered SMC-EPC-based vascular organoids were used for further experiments, including an *in vivo* study in a porcine MI model (Figure 1A).

### Cell characterization

Flow cytometry revealed a high purity of CD31^+^ cells, indicating the presence of EPCs (Figure 1B). Figure 1C shows the flow cytometry results used to characterize MSC-derived SMCs, which demonstrated a high purity of αSMA^+^/SM22α^+^ double-positive cells (99.8%). Flow cytometry analysis using calponin and caldesmon also showed double-positive cells with 93.1% purity.

### Surgical delivery of vascular organoids

Fourteen pigs were used in this study. Two were allocated to the sham group, which did not undergo MI creation. Two died during the first surgery (MI creation) due to ventricular fibrillation during ballooning to reach the LAD. One pig died two days after the first surgery, and another died during the second surgery. Therefore, only 4 pigs underwent the vascular organoid implantation. Implantation was performed under general anesthesia. Left mini-thoracotomy was performed, and the left ventricular (LV) wall of the heart was exposed (Figure 1D). Vascular organoids were successfully implanted in each pig. The pigs in the control group underwent left thoracotomy as in the experimental group under general anesthesia, and the chest was closed without implantation. Four pigs in each of the experimental and control groups survived and were followed up for four weeks after the second surgery.

### Vascular organoid transplantation improves function and slows dilated cardiomyopathy

The effects of vascular organoid transplantation on cardiac function were assessed in the porcine model using cardiac MRI and gadolinium delayed enhancement magnetic resonance imaging (DEMRI). Reference ranges were obtained from the Journal of Cardiovascular Magnetic Resonance.

**Figure 2:**
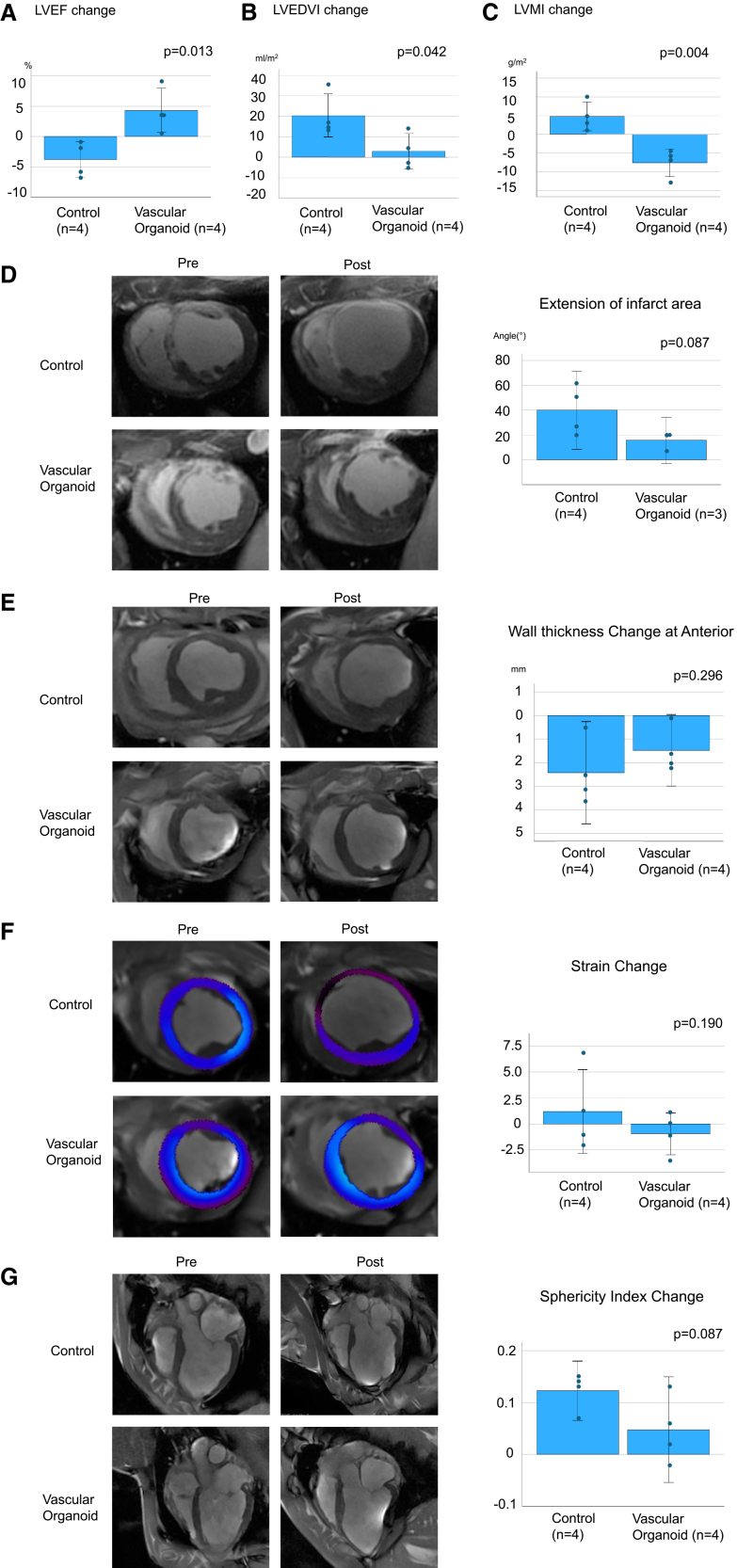
Cardiac magnetic resonance imaging (MRI) outcomes (A–C) Changes in left ventricular ejection fraction (LVEF) (A), left ventricular end-diastolic volume index (LVEDVI) (B), and left ventricular mass index (LVMI) (C) following treatment. (D) Representative delayed-enhancement MRI (DEMRI) images illustrate infarct area extension at the level of the papillary muscles. (E) Representative end-systolic MRI images and changes in wall thickness at the mid-anterior wall. (F) Functional assessment, including representative images of circumferential strain analysis. (G) Representative end-systolic MRI images of the left ventricle (LV) and changes in the sphericity index. Bars represent the mean ± standard error (SE), with each dot indicating an individual animal (*n* = 4 animals per group, except D, where *n* = 3 for the vascular organoid group). Student’s *t* test.

Left ventricular ejection fraction (LVEF) was similarly reduced below normal limits (reference range: ∼37.5%) in both the vascular organoid (*n* = 4) and control (*n* = 4) groups at post-IRI day 14 (experimental: 30.9 ± 1.9% vs. control: 28.2 ± 2.4%, *p* = 0.5). By post-IRI day 42, however, LVEF had significantly increased in the experimental group (+4.3 ± 1.7), whereas that in the control group exhibited a trend of continued worsening (−3.8 ± 1.7%) (*p* = 0.013) (Figure 2A). These changes led to a significantly different final LVEF (experimental: 35.2 ± 3.1% vs. control: 24.5 ± 2.5%, *p* = 0.01). The sham group (*n* = 2) had a normal LVEF (pre: 41.2%, post: 43.8%), and the mean change in LVEF over time was +2.6%, as expected.

Left ventricular end-diastolic volume index (LVEDVI), defined as LV end-diastolic volume/body surface area (BSA) (reference range: 47–107 mL/m^2^ vs. ∼74 mL/m^2^), was similarly elevated above normal limits in both groups at post-IRI day 14 (experimental, 110.2 ± 5.1 mL/m^2^ vs. control 113.7 ± 10.6 mL/m^2^, *p* = 0.8). By post-IRI day 42, LVEDVI had significantly increased in the control group (+20.4 ± 4.6 mL/m^2^) compared to the vascular organoid group (+2.5 ± 4.2 mL/m^2^, *p* = 0.042) (Figure 2B). The difference in final LVEDVI between both groups trended toward significance (experimental, 113.0 ± 6.0 mL/m^2^ vs. control, 134.1 ± 12.5 mL/m^2^, *p* = 0.053). As expected, the values in the sham group were 74.4 mL/m^2^ (pre) and 57.2 mL/m^2^ (post), and the mean change in LVEDVI was −17.2 mL/m^2^.

Left ventricular mass index (LVMI), defined as LV mass/BSA (reference range: 65–93 g/m^2^ vs. ∼64 g/m^2^), was similarly elevated in both groups at post-IRI day 14 (experimental, 99.1 ± 4.3 g/m^2^ vs. control, 99.4 ± 6.9). By post-IRI day 42, however, LVMI had significantly decreased in the experimental group (−7.6 ± 1.6 g/m^2^), whereas that in the control group significantly increased (+4.8 ± 1.6 g/m^2^) (*p* = 0.004) (Figure 2C). The mean change in LVMI in the sham group was −2.5 g/m^2^.

The extension of the infarct area was estimated using the angle of the infarct area on DEMRI. The angle at post-IRI day 14 was 140° (*p* = 0.970) in both groups. After treatment (day 42), it was 156° in the experimental group and 180° in the control group (*p* = 0.163), with changes of 16° and 40°, respectively (*p* = 0.087) (Figure 2D).

Wall thickness of the anterior wall at end-diastole was 5.25 mm and 5.55 mm in the vascular organoid and control groups at post-IRI day 14, respectively (*p* = 0.732). At post-IRI day 42, wall thickness was reduced in both groups, with changes of −2.43 ± 0.68 mm in the control group and −1.48 ± 0.48 mm in the experimental group (*p* = 0.296) (Figure 2E).

The circumferential strain in the anterolateral LV wall at the papillary muscle level was −7.9 and −8.6 in the experimental and control groups at post-IRI day 14, respectively (*p* = 0.772). Four weeks later, the values were −8.9 and −7.5, respectively (*p* = 0.624), with changes of −1.0 and 1.2 (*p* = 0.190) (Figure 2F).

The sphericity index values before thoracotomy were 0.62 and 0.63 in the vascular organoid and control groups (*p* = 0.908). After four weeks, the values were 0.68 and 0.75, respectively (*p* = 0.247), with changes of 0.123 ± 0.180 in the control group and 0.048 ± 0.320 in the experimental group (*p* = 0.087) (Figure 2G).

### Human-derived cells engraft and promote microangiogenesis

**Figure 3:**
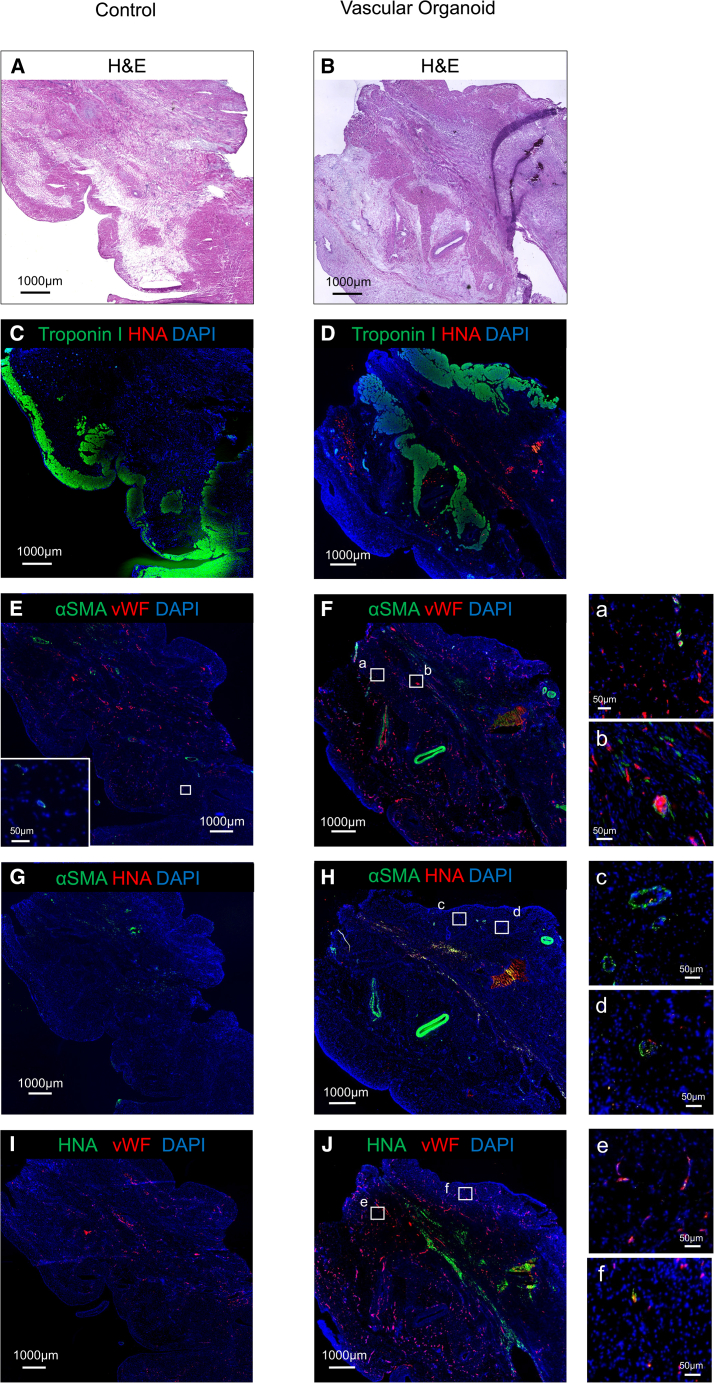
Histological and immunohistochemical (IHC) staining Representative images of the left ventricular border zone (*n* = 4 animals per group). (A and B) H&E staining of the border zone in the control group (A) and vascular organoid group (B). (C–J) Immunohistochemical staining in the control group (C, E, G, I) and vascular organoid group (D, F, H, J). (C, D) Troponin I and human nuclear antigen (HNA) staining; HNA-positive cells are visible in the vascular organoid group (D). (E and F) αSMA and vWF staining; αSMA^+^vWF^+^ cells were observed in the vascular organoid group (F), with high-magnification images of the boxed area shown in (a) and (b). (G and H) αSMA and HNA staining; αSMA^+^HNA^+^ cells were detected in pig heart (H), with high-magnification images in (c) and (d). (I and J) vWF and HNA staining; vWF^+^HNA^+^ cells indicate the migration of transplanted EPCs from the vascular organoid are shown in (J), with high-magnification images in (e) and (f).

Hematoxylin and eosin (H&E) and immunohistochemical (IHC) staining were performed for histological analysis. Figure 3 shows representative images of the LV border zone in the control and vascular organoid groups. H&E staining (Figures 3A and 3B) and troponin I staining (Figures 3C and 3D) demonstrated both troponin I-positive areas, indicating preserved myocardium, and troponin I-negative areas, indicating infarcted tissue, confirming the presence of a border zone.

Human nuclear antigen (HNA) staining revealed implanted cells in the vascular organoid group four weeks after surgery, whereas no HNA-positive cells were detected in the control group (Figures 3C and 3D). HNA^+^ cells were identified within the border zone of the pig heart tissue.

Figures 3E and 3F show αSMA and von Willebrand factor (vWF) staining. αSMA^+^/vWF^+^ cells indicated mature vasculature. αSMA^+^/HNA^+^ double-positive cells were observed in the vascular organoid group, suggesting the integration of implanted cells into the host myocardium, but were absent in the control group (Figures 3G and 3H). Similarly, HNA^+^/vWF^+^ double-positive cells were observed in the vascular organoid group, further indicating migration and incorporation of cells from the topically transplanted organoids into the host tissue (Figures 3I and 3J). In addition, HNA^+^ cells were identified within native vasculature, as shown in Figures 3C–3F.

### Microvascular density and maturation analysis

**Figure 4:**
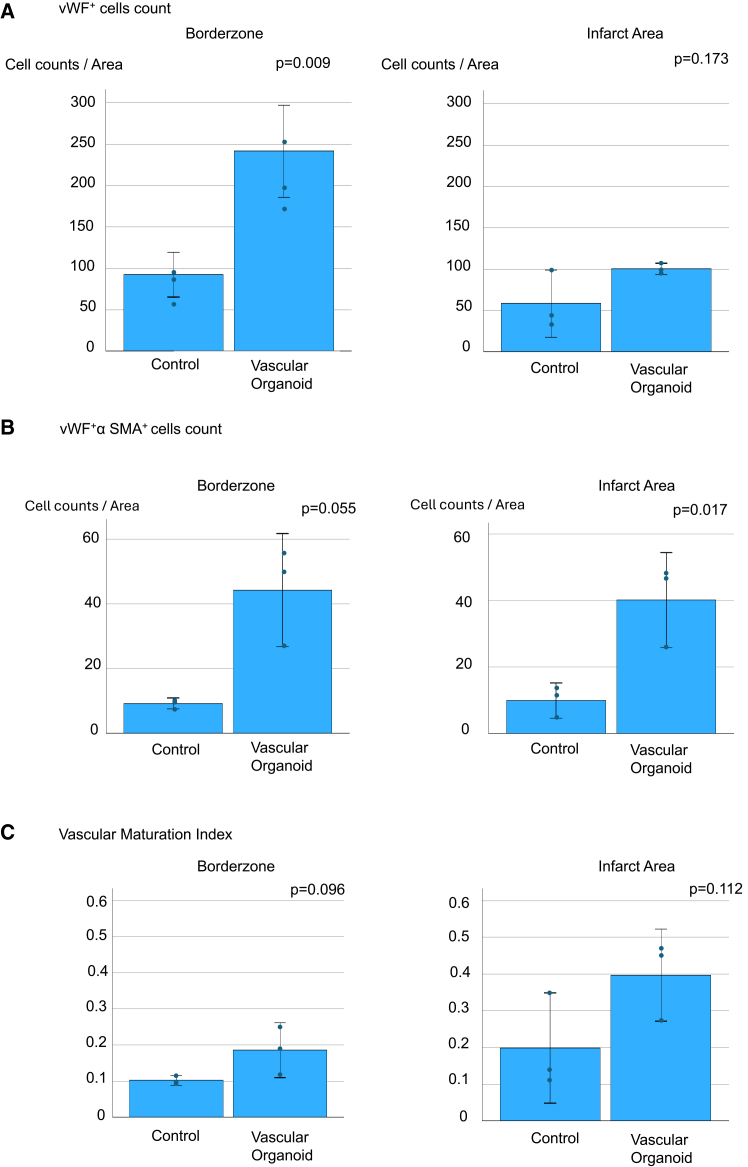
Microvascular density and maturation analysis (A) The number of vWF^+^ cells in the border zone and infarct area. (B) The αSMA^+^vWF^+^ cell counts in the border zone and infarct area. (C) The vascular maturation index, calculated as the ratio of αSMA^+^vWF^+^ cells to total vWF^+^ cells. Data are presented as the mean ± standard error (SE). *n* = 3 animals per group. Student’s *t* test.

IHC staining using vWF and αSMA revealed that the number of vWF^+^ cells in the border zone was significantly higher in the vascular organoid group than in the control group (241.5 ± 13.4 vs. 90.4 ± 9.5 cells/field, *p* = 0.009). A slight difference was observed in the infarct area (100.2 ± 10.9 vs. 58.4 ± 8.5 cells/field, *p* = 0.173) (Figure 4A). The ratio of HNA^+^/vWF^+^ double-positive cells among total vWF^+^ cells was 0.036 ± 0.015 in the vascular organoid group. In the border zone, the vascular organoid group demonstrated higher αSMA^+^vWF^+^ cell counts than the control group (44.3 ± 9.5 vs. 8.3 ± 2.2 cells/field, *p* = 0.055), and this tendency was also observed in the infarct area (40.1 ± 8.5 vs. 9.9 ± 3.3 cells/field, *p* = 0.017) (Figure 4B).

The vascular maturation index, calculated as the ratio of αSMA^+^vWF^+^ cells to total vWF^+^ cells, was higher in the vascular organoid group (Figure 4C). In the border zone, the maturation index was 0.191 ± 0.035 vs. 0.102 ± 0.025 (*p* = 0.096), and in the infarct area, 0.365 ± 0.040 vs. 0.174 ± 0.053 (*p* = 0.112).

### Differential gene expression analysis reveals significant transcriptional changes in the mid-border zone following EPC-SMC vascular organoid treatment

**Figure 5:**
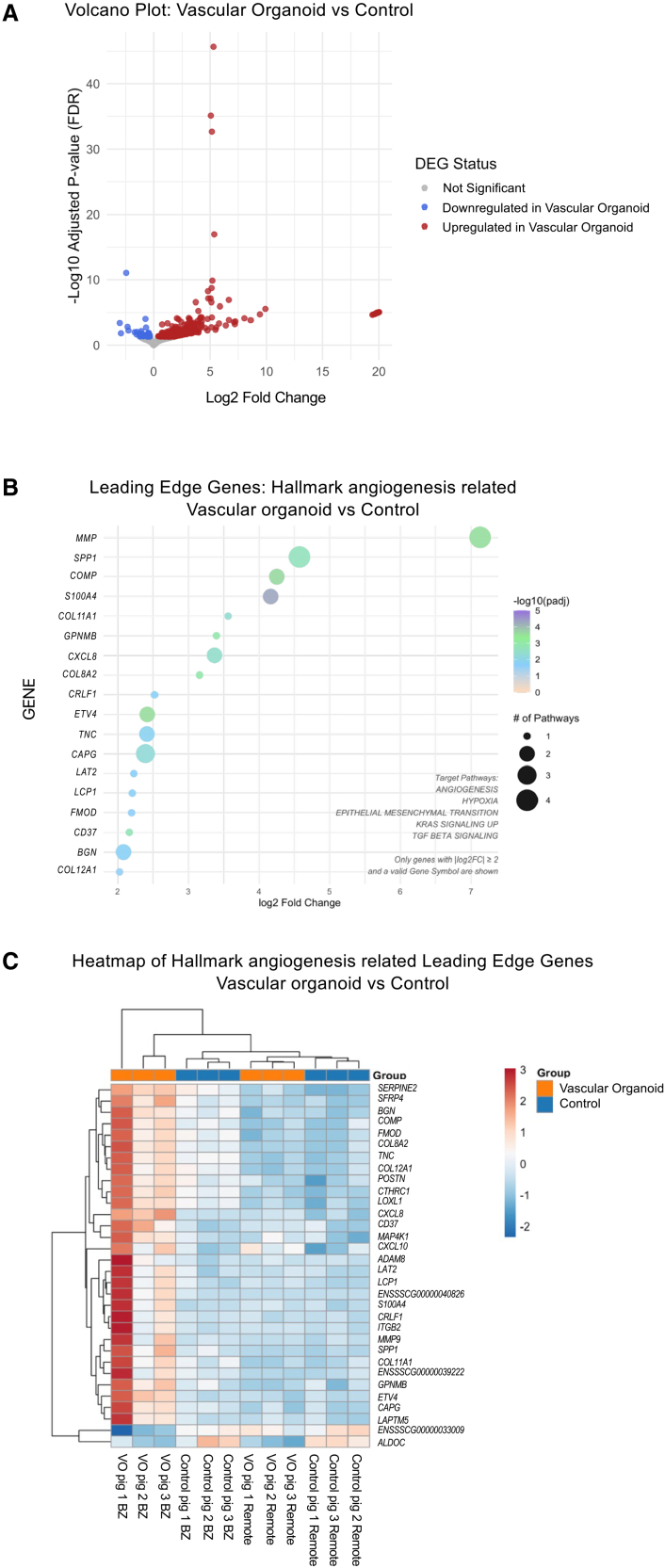
Differential gene expression and pathway analysis (A) Results of differentially expressed gene analysis using RNA-sequencing data are summarized in a volcano plot. (B) Hallmark angiogenesis-related leading-edge genes (LEGs); fold change of enriched genes in the vascular organoid group compared to controls is shown. (C) Heatmap of angiogenesis-related LEGs. *n* = 3 animals per group. BZ, border zone. VO, vascular organoid.

To identify the specific genes regulated by topically transplanted SMC-EPC-based vascular organoids within the ischemic border zone, we analyzed differentially expressed genes (DEGs) using RNA-seq data from mid-border zone samples of the experimental and control groups (*n* = 3 animals per group). A total of 400 DEGs (adjusted *p* value <0.05) were identified, with 349 upregulated and 51 downregulated in the vascular organoid-treated group compared to those in the control group. The results were summarized in a volcano plot (Figure 5A).

### Gene set enrichment analysis (GSEA) highlights angiogenesis-related transcriptional programs in the border zone

To explore the functional relevance of the transcriptional changes observed in the mid-border zone, we performed GSEA using the MSigDB Hallmark gene set. Among the analyzed pathways, epithelial-mesenchymal transition and KRAS signaling demonstrated the strongest enrichment signals (|NES| > 2, FDR < 0.05). Hypoxia and angiogenesis also exhibited positive enrichment (|NES| > 1.5, FDR < 0.05).

Based on these enriched pathways, we extracted the leading-edge genes (LEGs) and visualized their expression profiles (Figure 5B). Many angiogenesis-related genes were consistently upregulated in the experimental border zone samples, with several genes participating in multiple enriched pathways.

A heatmap (Figure 5C) confirmed consistent upregulation of signature genes in the experimental border zone samples, whereas other regions, including the remote myocardium, showed no notable differences. These data reinforce that the transcriptional effects of vascular organoid therapy are localized to the ischemic border zone and specifically involve angiogenesis-related gene programs.

## Discussion

Although angiogenic therapies for IHF remain highly sought after, traditional cell delivery platforms, such as direct myocardial injection or systemic administration, have demonstrated limited success. These approaches often suffer from poor localization, low cell retention, and risk of adverse outcomes, including arrhythmogenic foci or ectopic tissue formation (Dohmann et al., 2005; Van Den Akker et al., 2017). Topically applied therapies address many of these limitations by avoiding direct myocardial trauma or systemic dispersion; however, they also come with challenges, such as potentially restricting myocardial compliance and contributing to constrictive diastolic dysfunction. Moreover, it remains unclear whether epicardial patch-based therapies relying on passive diffusion can achieve therapeutic benefits across thick myocardial walls.

To address these limitations, we evaluated topically transplanted SMC-EPC-based vascular organoids as an adjunct therapy for IHF, particularly in cases driven by microvascular malperfusion. We used a porcine model of IRI to replicate the clinical context of a large anterolateral MI followed by coronary revascularization using procedures such as CABG or PCI. Prior studies using this model have shown a rapid decline in LVEF and an increase in LVEDV during the first two weeks post-injury, highlighting the critical role of microvascular malperfusion in disease progression (McCall et al., 2012; Oduneye et al., 2015). We therefore selected a 14-day latency period before vascular organoid transplantation to coincide with the maturation phase of myocardial wound healing.

The organoid employed in this study was a scaffold-free, compliant construct designed to accommodate the dynamic motion of the heart without imposing mechanical restrictions. Geometric and strain analyses confirmed the absence of restrictive physiology and suggested the attenuation of progressive remodeling toward dilated cardiomyopathy. Cardiac MRI further supported these findings, demonstrating the preservation of LVMI with only a mild increase in LVEDVI.

Global LV function was significantly improved in the treatment group. Specifically, animals receiving vascular organoid therapy exhibited an increase in LVEF of over 4% from baseline, despite concurrent administration of tacrolimus, an immunosuppressive agent known to impair myocardial performance (Kise et al., 2010). Moreover, previous studies have suggested that an LVEF increase exceeding 4% may correlate with improved survival outcomes (Roe et al., 2010).

When examining regional function, circumferential strain analysis revealed a favorable trend in the treatment group, whereas the controls exhibited progressive deterioration. These findings support the hypothesis that vascular organoids alleviate microvascular malperfusion, particularly within the border zone, thereby preserving hibernating cardiomyocytes and sustaining regional myocardial function. Consistent with this mechanism, RNA sequencing revealed the upregulation of angiogenesis-related pathways, and not only HNA^+^ human-derived vessels but also host-derived vasculature was observed, suggesting paracrine effects on the host border zone—features characteristic of hibernating myocardium (Rahimtoola, 1989).

Changes in LV geometry, particularly increased sphericity, are recognized as hallmarks of adverse remodeling during heart failure progression (Wu et al., 2024). While no statistically significant differences were detected between groups, the sphericity index increased over time in both groups, with the vascular organoid group exhibiting a slower rate of increase, suggesting a possible trend toward reduced geometric deterioration.

To address whether topically applied therapies can achieve meaningful benefits across thick myocardial tissue, we used porcine IRI, which closely recapitulates the myocardial wall thickness of human hearts. Rather than canonical hypoxia-inducible pathways, gene expression analysis revealed strong upregulation of genes associated with endothelial and SMC proliferation, consistent with the 42-day recovery period during which normoxia was likely restored.

GSEA confirmed robust activation of angiogenesis-related transcriptional programs in the vascular organoid-treated group. The upregulated LEGs, including *MMP9, SPP1, COMP, CXCL8, S100A4*, and *COL11A1*, are known to promote extracellular matrix remodeling, endothelial migration, and neovessel formation. Importantly, many of these gene programs appear to result from the synergistic interactions between EPCs and SMCs within organoids. *SPP1* is predominantly secreted by SMCs and facilitates EPC adhesion (Liaw et al., 1994), *MMP9* expression is enhanced when both cell types are present (Waltera et al., 2023), and *COMP* contributes to vascular structural integrity (Jia et al., 2018). Co-culture of EPCs and SMCs also enhances *PI3K-AKT* signaling (Brown et al., 2005), a pathway upregulated in the treated myocardium.

Histological analyses demonstrated higher numbers of vWF^+^ endothelial cells and αSMA^+^vWF^+^ arterioles in treated hearts, along with an increased vascular maturation index. Given the very low HNA^+^/vWF^+^ ratio, the majority of these arterioles are considered host-derived, suggesting a paracrine effect of the topically transplanted vascular organoids (Shudo et al., 2013, 2017). Transmission electron microscopy suggested that mitochondrial morphology was relatively preserved in treated hearts (data not shown).

Our study has several limitations. The use of a 2-week delayed transplantation model may miss early therapeutic windows. The observation period was limited to one month, which may not fully capture long-term outcomes. Moreover, tacrolimus is a known angiogenesis inhibitor that may have partially masked the full pro-angiogenic effect. Despite immunosuppression, the potential for xenogeneic rejection remains.

In conclusion, our study provides early proof of concept for topically transplanted SMC-EPC-based vascular organoids as a novel interventional therapy for IHF driven by microvascular malperfusion. This approach attenuates the progression of both dilated and constrictive heart failure phenotypes, improves cardiac function, and preserves myocardial geometry. The use of clinically translatable components further strengthens the therapeutic potential of this strategy.

## Resource availability

### Lead contact

Requests for further information and resources associated with this study should be directed to lead contact Yasuhiro Shudo (yshudo@stanford.edu).

### Materials availability

Materials used in this study are available from the lead contact, Yasuhiro Shudo, upon request.

### Data and code availability

• RNA sequencing data have been deposited in NCBI GEO and are publicly available as of the date of publication. The accession number is GSE333973 and is also listed in the key resources table.
• All other data reported in this paper will be shared by the lead contact upon reasonable request.
• This paper does not report original code.

## Acknowledgments

This study was supported by the American Heart Association Career Development Award 23CDA1038803 and the 10.13039/100019607Stanford Cardiovascular Institute / Steven M. Gootter Foundation 2020 Seed Grant.

## Author contributions

Conceptualization: J.F. and Y.S.; methodology: J.F., S.Y., E.P., U.S., T.U., H.T., Y.M., G.I., and Y.L.; investigation: J.F., S.Y., Y.K., K.K., E.P., U.S., J.L., T.U., H.T., Y.M., Y.Z., and S.E.; writing: J.F., Y.K., and K.K.; reviewing and editing: Y.K. and Y.S.; supervision: P.Y. and J.W.

## Declaration of interests

The authors declare no competing interests.

## STAR★Methods

### Key resources table

**Table:** 

REAGENT or RESOURCE	SOURCE	IDENTIFIER
**Antibodies**		

anti-Human Nuclear Antigen antibody	Abcam	Cat: ab191181; 1:100; RRID: AB_2885016
anti-Cardiac Troponin I antibody	Abcam	Cat: ab47003; 1:100; RRID: AB_869982
anti-von Willebrand factor (vWF)	Abcam	Cat: ab11713; 1:100; RRID: AB_298501
anti-alpha smooth muscle Actin (αSMA)	Abcam	Cat: ab240654; 1:100; RRID: AB_2922779
Donkey anti-Rabbit IgG (secondary)	Abcam	Cat: ab150073; 1:200; RRID: AB_2636877
Donkey anti-Mouse IgG (secondary)	Abcam	Cat: ab150107; 1:200; RRID: AB_2890037
Donkey anti-Sheep IgG (secondary)	Abcam	Cat: ab150177; 1:200; RRID: AB_2801320
Goat anti-Mouse IgG (secondary)	Abcam	Cat: ab150116, ab150179; 1:200; RRID: AB_2688012

**Biological Samples**		

Human peripheral blood (EPC source, healthy female donors)	STEMCELL Technologies	Vancouver, Canada
Human bone marrow-derived mesenchymal cells (SMC source)	Lonza, Inc.	NJ, USA
Yorkshire pig cardiac tissue	This study	N/A

**Chemicals, Peptides, and Recombinant Proteins**		

EGM-2 BulletKit	Lonza Inc.	Cat: CC-3162
MEΜα medium	Life Technologies	CA, USA
SmGM-2 BulletKit	Lonza, Inc.	NJ, USA
TGF-β	Sigma-Aldrich	St. Louis, MO; 2 ng/μL
Human plasma fibronectin	Sigma-Aldrich	Coating for dishes
TISSEEL fibrin sealant	Baxter Laboratories, Inc.	Glennview, IL, USA
Tacrolimus	N/A	0.75 mg/kg/day
Mycophenolate mofetil	N/A	500 mg/day
Prednisolone	N/A	20 mg/day
Lymphoprep	STEMCELL Technologies	Vancouver, Canada
OCT compound	Fisher HealthCare	Cat: 23730571

**Critical Commercial Assays**		

EZ1 RNA Tissue Mini Kit	QIAGEN	Hilden, Germany
Upcell dish (temperature-responsive)	CellSeed	Tokyo, Japan

**Deposited Data**		

RNA sequencing data	This study	NCBI GEO: GSE333973

**Experimental Models: Organisms/Strains**		

Yorkshire pig (Sus scrofa, female, 10–12 weeks, 35–50 kg)	Premier BioSource, Inc.	N/A

**Software and Algorithms**		

DESeq2 v1.48.0	Bioconductor	RRID: SCR_015687
STAR v2.7.10a	Dobin et al., 2013	RRID: SCR_004463
featureCounts v2.16.1	Liao et al., 2014	RRID: SCR_012919
GSEA v1.70.1	Broad Institute	RRID: SCR_003199
MSigDB Hallmark gene set v7.5.1	Broad Institute	RRID: SCR_016863
R v4.5.0	R Core Team	RRID: SCR_001905
SPSS v29	IBM	RRID: SCR_002865
ggplot2	Wickham, 2016	RRID: SCR_014601
FastQC v0.12.1	Babraham Bioinformatics	RRID: SCR_014583
MultiQC v1.17	Ewels et al., 2016	RRID: SCR_014982
Keyence BZ-X800 software	Keyence	Osaka, Japan

### Experimental model and study participant details

#### Animal model

Female Yorkshire pigs (Sus scrofa, 10–12 weeks old, 35–50 kg) were obtained from Premier BioSource, Inc. All animals were socially housed in a temperature-controlled room at approximately 22°C with a 12 h light/dark cycle. Food and water were provided *ad libitum*. All animal experiments were conducted in compliance with the Guide for the Care and Use of Laboratory Animals (8th Edition, National Research Council, 2011) and adhered to the ARRIVE guidelines. The study protocol was approved by the Stanford University Administrative Panel on Laboratory Animal Care (APLAC) under Protocol Number APLAC-34046.

#### Human cell sources

Human EPCs were isolated from peripheral blood of healthy female donors (STEMCELL Technologies, Vancouver, Canada). Human bone marrow-derived mesenchymal cells were obtained from a commercial source (Lonza, Inc., NJ, USA). All cell use was in accordance with institutional guidelines.

### Method details

#### EPC isolation and culture

Human EPCs were isolated from peripheral blood of healthy female donors using density gradient centrifugation. Mononuclear cells were collected by layering blood over Lymphoprep (STEMCELL Technologies) and centrifuging at 560 × g for 30 min. Collected cells were washed in PBS and resuspended in EGM-2 BulletKit (Cat. CC-3162; Lonza Inc., NJ, USA). Isolated EPCs were seeded on 6-well plates coated with fibronectin at 50 × 10^6^ cells/well and cultured at 37°C, 5% CO_2_. Media were exchanged every 24 h for the first seven days and every 48 h thereafter. Colonies exhibiting EPC cobblestone morphology appeared on days 10–14 and reached confluence by days 24–28. After the first passage, cells were cultured on 0.2% gelatin. Cells at passages 4–9 were used for assays and *in vivo* implantation. Flow cytometry for CD31 was performed to confirm cell characterization (Masouleh et al., 2010).

#### SMC modulation and culture

Human bone marrow-derived mesenchymal cells were cultured in MEΜα medium (Life Technologies, CA) supplemented with 10% FBS, 1% GlutaMAX (Gibco), and gentamicin at 37°C, 5% CO_2_. For conversion to the SMC phenotype, cells at approximately 85% confluency were plated in dishes coated with human plasma fibronectin in SmGM-2 BulletKit (Lonza, Inc., NJ) supplemented with 2 ng/μL TGF-β (Sigma-Aldrich, St. Louis, MO) (Shin et al., 2022). SMCs at passages 5–10 were characterized by αSMA, SM22α, and caldesmon immunostaining (Shudo et al., 2016; Shin et al., 2022; Kawamura et al., 2017).

#### Vascular organoid fabrication

Vascular organoids were fabricated using temperature-responsive Upcell dishes (CellSeed, Tokyo, Japan) coated with poly(N-isopropylacrylamide). A 100 mm Upcell dish was conditioned in pre-warmed 231 medium for 30 min at 37°C. Then, 1.0 × 10^7^ human SMCs were seeded and cultured for 24 h at 37°C. Next, 1.0 × 10^7^ EPCs suspended in EGM-2 medium were carefully seeded on top of the confluent SMC monolayer and cultured for an additional 24 h. The dish was then transferred to room temperature (20°C–22°C), allowing the bilayer to detach as an intact 45 mm diameter scaffold-free EPC-SMC vascular organoid (Shin et al., 2022; Kawamura et al., 2017).

#### Porcine IHD model creation

Yorkshire pigs (10–12 weeks old) were fasted for 12 h and anesthetized with ketamine (5–15 mg/kg IM) and midazolam (0.1–0.5 mg/kg IM), endotracheally intubated, and maintained on 1–3% inhaled isoflurane. Buprenorphine (0.27 mg/kg SQ) and carprofen (5 mg/kg) were administered for analgesia. Cefazolin (25–35 mg/kg IV) was given as prophylactic antibiotic. A catheter was inserted via the right common carotid artery using the Seldinger technique. The left anterior descending coronary artery (LAD) was identified by fluoroscopy, and a balloon was inserted and inflated for 60 min to induce ischemia-reperfusion injury (IRI). After IRI, formal left coronary angiography confirmed the absence of residual large vessel filling defects. Animals were extubated and recovered in standard housing for 14 days (McCall et al., 2012). Starting on post-IRI day 9, animals in both groups were administered immunosuppressants: tacrolimus (0.75 mg/kg/day), mycophenolate mofetil (500 mg/day), and prednisolone (20 mg/day). Doses were adjusted to achieve target tacrolimus levels of 10–20 ng/mL during week 1 and 5–15 ng/mL during weeks 2–4.

#### Surgical delivery of vascular organoids

On post-IRI day 14, animals underwent a 5 cm left anterolateral thoracotomy at the 4th intercostal space. The sham group (*n* = 2) underwent thoracotomy without prior IRI. In the experimental group (*n* = 4 animals), vascular organoids (10 per animal) were placed over the entire infarct and surrounding border zones with the EPC side facing the myocardium and secured with TISSEEL fibrin sealant (Baxter Laboratories, Inc., Glennview, IL). In the control group (*n* = 4 animals), only TISSEEL was applied. Animals were randomly assigned to each group. Immunosuppression was continued until euthanasia on post-thoracotomy day 28.

#### Cardiac MRI

Cardiac MRI was performed on post-IRI day 14 (pre-implantation) and post-IRI day 42 (28 days after thoracotomy) using a Signa 3 T EXCITE scanner (GE Healthcare, WI, USA) with a phased array 4-channel surface coil. ECG-gated fast-spoiled gradient-recalled (FSPGR) sequences were used to evaluate LV function. DEMRI was performed using IR-FSPGR at TI 250 ms following IV injection of 0.2 mmol/kg Magnevist (Bayer NJ) (Dash et al., 2011, 2015; Tada et al., 2019). LV contours were traced manually to calculate LVEDV, LVESV, and LVEF. Infarct extension, wall thickness, circumferential strain, and sphericity index were also assessed. MRI analysis was performed blinded.

#### Tissue harvest and RNA sequencing

Hearts were harvested four weeks after thoracotomy. Samples from infarct, border, and remote zones were collected for RNA sequencing and histology. Tissues for the infarct area were collected according to visual appearance; those for the border zone were determined as the section adjacent to the infarct area; and those for the remote zone were collected from the posterior wall. Tissue for RNA sequencing was immediately flash-frozen in liquid nitrogen and stored at −80°C.

Total RNA was extracted using the EZ1 RNA Tissue Mini Kit (QIAGEN, Hilden, Germany). All 12 samples passed quality assessment. Libraries were prepared using poly(A) enrichment (nondirectional) and paired-end sequencing (150 bp) was performed on the Illumina NovaSeq X Plus platform (Novogene, Sacramento, CA). Raw reads were assessed with FastQC (v0.12.1) and MultiQC (v1.17), aligned to Sus scrofa (Sscrofa11.1, Ensembl release 113) using STAR (v2.7.10a), and quantified using featureCounts (v2.16.1). Only high-quality border zone samples (*n* = 3 animals per group) were included in downstream analyses. One animal was excluded from each group: the control group sample was improperly stored, and the experimental group sample was excluded due to infection. Differential gene expression analysis was performed using DESeq2 (v1.48.0) with adjusted *p*-value <0.05. GSEA (v1.70.1) was performed against the MSigDB Hallmark gene set (H collection, v7.5.1) (Liberzon et al., 2015). RNA sequencing was performed blinded.

#### Immunohistochemical staining and assessment of mature vessel formation

Mid border zone samples were cryo-sectioned at 10 μm thickness and stained with anti-Human Nuclear Antigen antibody (1:100, Abcam; Cat: ab191181), anti-Cardiac Troponin I antibody (1:100, Abcam; Cat: ab47003), anti-vWF (1:100, Abcam; Cat: ab11713), and anti-αSMA antibody (1:100, Abcam; Cat: ab240654). Secondary antibodies (1:200, Abcam; Cat: ab150073, ab150107, ab150117, ab150116, ab150179) were used, and nuclei were counterstained with DAPI. Images were acquired using a Keyence BZ-X800 microscope (Osaka, Japan). Microvascular density was calculated using Keyence analysis software by identifying the number of αSMA^+^/vWF^+^ arterioles per 20× image field from five randomly selected fields per slide (*n* = 4 animals per group for vWF^+^ counts; *n* = 3 animals per group for vascular maturation index). The vascular maturation index (VMI) was calculated as the ratio of αSMA^+^vWF^+^ cells to total vWF^+^ cells (Kainuma et al., 2015; Bhattacharya et al., 2008). IHC staining was performed blinded; cell counting was performed automatically.

### Quantification and statistical analysis

Data are presented as mean ± SE. Normality was assessed using the Shapiro–Wilk test and probability plots. For group comparisons, Student’s *t* test (parametric) was used. Statistical significance was defined as *p* < 0.05. Analyses were conducted using R (v4.5.0; R Core Team, 2023) and SPSS v29; plots were generated with the 'ggplot2' package (Wickham, 2016). Sample sizes are indicated in individual figure legends. For Figure 4, Student’s *t* test was applied to compare vascular organoid and control groups within each anatomical region (border zone and infarct area) independently.

### Additional resources

Further information and requests for resources and reagents should be directed to and will be fulfilled by the lead contact, Yasuhiro Shudo (yshudo@stanford.edu).
